# Principles of Charge Estimation Methods Using High-Frequency Current Transformer Sensors in Partial Discharge Measurements

**DOI:** 10.3390/s20092520

**Published:** 2020-04-29

**Authors:** Armando Rodrigo-Mor, Fabio A. Muñoz, Luis Carlos Castro-Heredia

**Affiliations:** Electrical Sustainable Energy Department, Delft University of Technology, 2628 CD Delft, The Netherlands; f.a.munozmunoz-1@tudelft.nl (F.A.M.); L.C.CastroHeredia@tudelft.nl (L.C.C.-H.)

**Keywords:** high-frequency current transformer, HFCT, Rogowski coils, magnetic loop antenna, partial discharge, PD, magnetically coupled sensors, charge estimation

## Abstract

This paper describes a simplified model and a generic model of high-frequency current transformer (HFCT) sensors. By analyzing the models, a universal charge estimation method based on the double time integral of the measured voltage is inferred. The method is demonstrated to be valid irrespective of HFCT sensor, assuming that its transfer function can be modelled as a combination of real zeros and poles. This paper describes the mathematical foundation of the method and its particularities when applied to measure nanosecond current pulses. In practice, the applicability of the method is subjected to the characteristics and frequency response of the sensor and the current pulse duration. Therefore, a proposal to use the double time integral or the simple time integral of the measured voltage is described depending upon the sensor response. The procedures used to obtain the respective calibration constants based on the frequency response of the HFCT sensors are explained. Two examples, one using a HFCT sensor with a broadband flat frequency response and another using a HFCT sensor with a non-flat frequency response, are presented.

## 1. Introduction

Partial discharge (PD) measurements are a fundamental tool for insulation diagnostics. The conventional method, defined in the standard IEC 60270 [1], presents the concept of apparent charge. Moreover, it sets the procedure for charge calibration and the bandwidths for charge estimation. As presented in [2], the charge estimation method defined in the IEC 60270 is based on the impulse response of bandpass filters—the bandwidth of which is stated in the standard.

On the other hand, there is a variety of unconventional partial discharge measurements that rely on high-frequency current transformer (HFCT) sensors, Rogowski coils and magnetic sensors [3,4,5,6,7,8,9]. Recently, it has been shown that HFCT can also be used in gas-insulated systems (GISs) [10]. Alternative charge estimation methods are required if the bandwidth of the HFCT sensor does not fall within the IEC60270 frequency ranges or if it is needed to resolve the PD pulses in the μs range. As described in [2], several techniques are available for charge estimation methods in unconventional partial discharge measurements. One of the proposed charge estimation techniques is based on the frequency domain analysis, while others rely on the evaluation of the time integral of the measured voltage. The method based on the evaluation of the time integral of the measured voltage is suitable when the HFCT sensor has a flat and broadband frequency response. However, if the HFCT sensor has a non-flat frequency response, then the application of the time integral of the measured voltage leads to significant charge estimation errors. Therefore, a charge estimation method is needed for HFCT sensors with a non-flat frequency response.

This paper shows a simplified and a generic model that describes the frequency response of a HFCT. Using the generic model, a basic principle of charge estimation based on the evaluation of the double time integral of the measured voltage is inferred. This method is proven to be theoretically suitable irrespective of the HFCT frequency response. However, its practical application depends on the required integration time, which is related to the current pulse duration and the frequency response of the sensor.

Finally, two examples are presented. The first one uses a HFCT sensor with a non-flat frequency response where the charge of a current pulse is estimated using the double time integral method. In the second example, a HFCT sensor with a broadband flat frequency response is used along with the time integral of the measured voltage. The calculation of the calibration constants for each sensor and method is also explained. 

## 2. Equivalent Model of HFCT Sensors

### 2.1. Simplified HFCT Model

**Figure 1:**
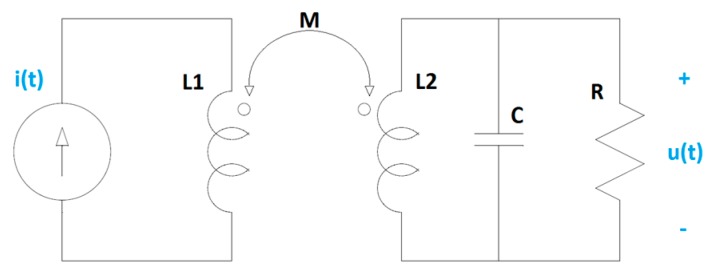
Electric circuit model of a magnetically coupled sensor.

HFCT sensors can be modelled as magnetically coupled coils. Figure 1 shows a simplified electric circuit model, where *L*_1_ is the self-inductance of the primary coil, *L*_2_ is the self-inductance of the secondary coil, *M* is the mutual inductance, *C* represents the parasitic capacitance of the secondary coil and *R* is a loading resistor. 

For the above electric circuit model, the differential equation that describes the relationship between the input, *i*(*t*), and the output, *u*(*t*), can be written as in Equation (1), being *M* = *k·√*(*L*_1_*·L*_2_) and *k* the coupling coefficient.

(1) $$\frac{di\left(t\right)}{dt}=\frac{L_{2}C}{M}\frac{d^{2}u\left(t\right)}{dt^{2}}+\frac{L_{2}}{RM}\frac{du\left(t\right)}{dt}+\frac{u\left(t\right)}{M}$$

In the Laplace domain, the transfer function of the sensor is described by the following equation.

(2) $$H\left(s\right)=\frac{U\left(s\right)}{I\left(s\right)}=\frac{s}{\frac{L_{2}C}{M}s^{2}+\frac{L_{2}}{RM}s+\frac{1}{M}}$$

If *R < 0.5·√*(*L*_2_*/C*), the transfer function of the sensor, as modelled by Equation (1), can be rewritten as a generic transfer function with real zeros and poles as follows.

(3) $$H\left(s\right)=\frac{U\left(s\right)}{I\left(s\right)}=\frac{\alpha \cdot s}{\left(s+p_{1}\right)\left(s+p_{2}\right)}$$

where *p*_1_ and *p*_2_ are poles representing the lower and upper cutoff frequencies of the sensor, and α is a constant.

The frequency response *H*(ω) of the sensor can be written as in Equation (4), where ω is the angular frequency.

(4) $$H\left(\omega \right)=\frac{U\left(\omega \right)}{I\left(\omega \right)}=\frac{j\omega M}{\left(1-L_{2}C\omega^{2}\right)+j\omega \frac{L_{2}}{R}}\;\left[\frac{V}{A}\right]$$

According to Equation (4), the maximum sensor gain occurs at frequency *f_o_* and accounts for *H_max_*, as described in the following equations.

(5) $$f_{o}=\frac{1}{2\pi \sqrt{L_{2}\cdot C}}$$

(6) $$H_{max}=\frac{R\cdot M}{L_{2}}\;\left[\frac{V}{A}\right]$$

This simplified model of HFCT sensors, and, in general, of any sensor based on magnetically coupled inductors, describes the sensor response when the lower and upper cutoff frequencies are separated enough, which means that the poles do not interfere with each other.

**Figure 2:**
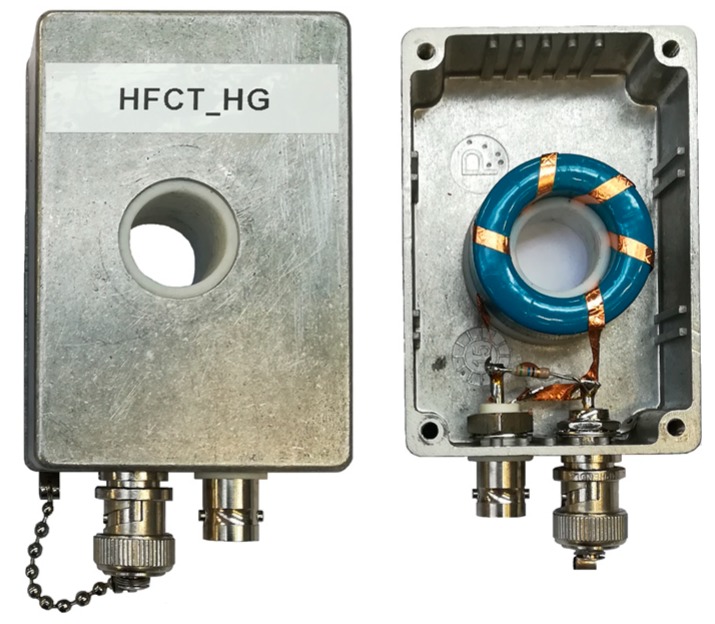
High-frequency current transformer (HFCT) enclosure and detailed inner view.

To illustrate this behavior, a HFCT sensor based on a N30 magnetic material core has been characterized; see Figure 2. The sensor has a 5-turn secondary winding, made with a flat copper ribbon. The sensor is loaded with a resistor, R, in parallel with a ceramic capacitor, C. The values of R and C are shown in Table 1. The terminals of the secondary coil are connected to an isolated BNC connector. The shield of the isolated BNC connector is connected to the central conductor of a BNC connector—the shield of which is connected to the HFCT enclosure. By using a shorted BNC cap, the terminal of the coil is connected to the enclosure. A teflon ring is used to keep the magnetic core in place. The whole arrangement is in a metallic enclosure for electric shielding purposes. Table 1 shows the parameters of the sensor named HFCT_LG.

**Figure 3:**
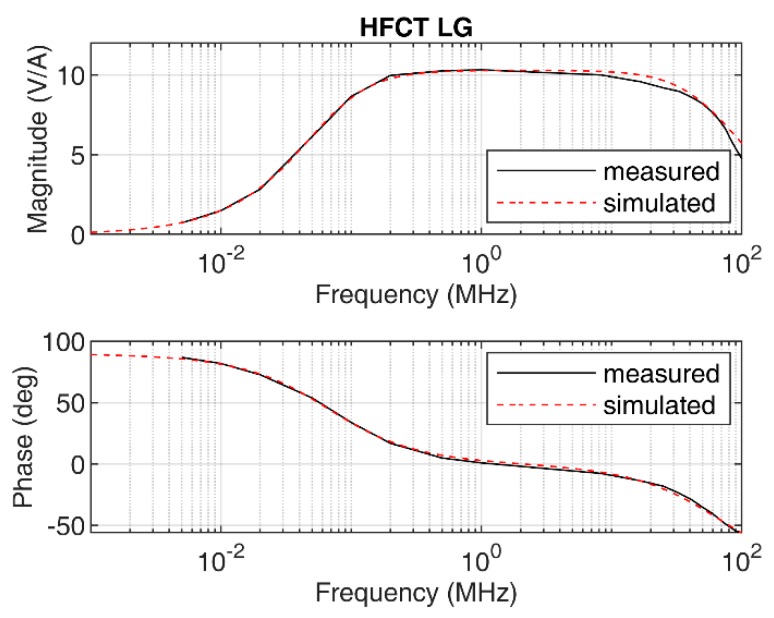
HFCT_LG sensor frequency response, measured and simulated.

The sensor has a flat response, with a maximum gain of 10.3 mV/mA and a bandwidth from 67 kHz to 67 MHz; see Figure 3. Using the measured frequency response, the two poles *p_1_* and *p_2_*, 2π∙67 × 10^3^ rad/s and 2π∙67 × 10^6^ rad/s, respectively, are estimated. The poles correspond to the lower and upper cutoff frequencies, 67 kHz and 67 MHz, correspondingly. The value of α is set to 4.34 × 10^9^. The simulated frequency response obtained using Equation (4) is depicted in Figure 3 along with the measured frequency response. The figure shows the good agreement between the measurement and the simulation, which validates the simplified model for this particular sensor.

### 2.2. Generic HFCT Model

In some situations, the frequency response of a sensor based on magnetically coupled inductors cannot be represented by the simplified model, particularly when the sensor does not exhibit a flat and wide frequency response. The deviations from the simplified model are due to different aspects not taken into account in the simplified model. Some of them are the permeability frequency dependence behavior, the non-linear losses in the magnetic core, the multiple coil resonances or, for instance, the effect of parasitic capacitances. 

To illustrate this fact, another sensor has been built using different resistor values while using the same magnetic core material and number of turns in the secondary coil as the previous sensor. Table 2 shows the parameters of the sensor named HFCT_HG. 

**Figure 4:**
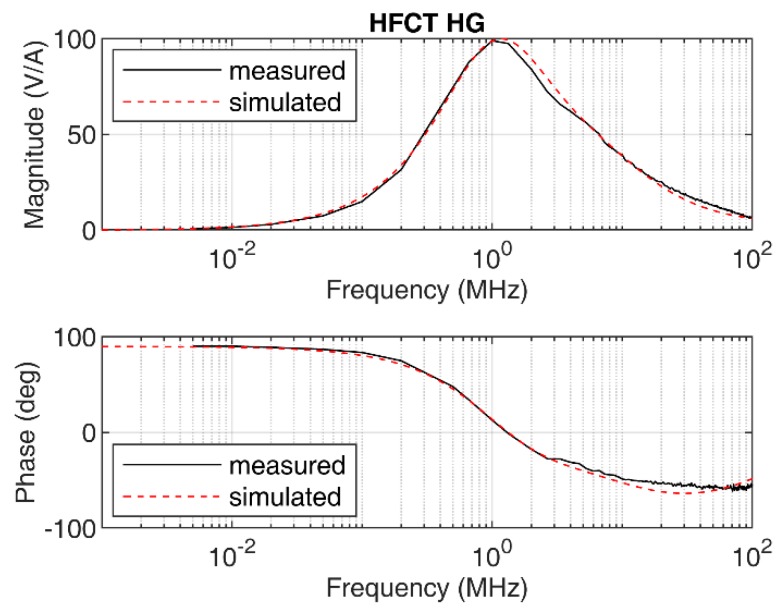
HFCT_HG sensor frequency response, measured and simulated.

The measured frequency response of the sensor is shown in Figure 4. Due to the parameters of the sensor, its frequency response exhibits a peak response at approximately 1 MHz, with 100 mV/mA peak gain. 

**Figure 5:**
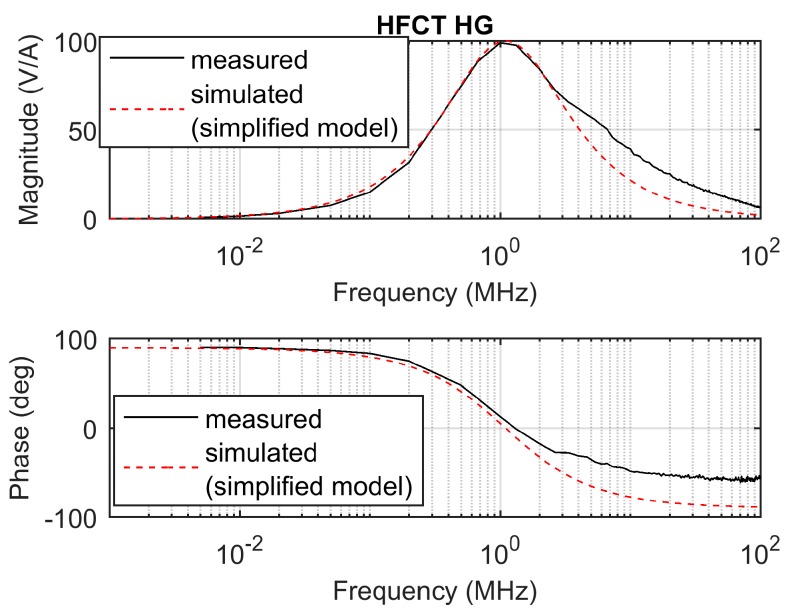
HFCT_HG sensor frequency response, measured and simulated, using the simplified model (one zero and two poles).

In this case, the sensor cannot be modelled by the simplified model, since the shape of the measured frequency response does not match with the transfer function shown in Equation (3). This is illustrated in Figure 5, where a simulated frequency response according to Equation (4) indicates a proper fit in the low-frequency range but fails for frequencies above 1 MHz in both the magnitude and the phase frequency response. 

In order to obtain a good fitting model, the number of zeros and poles must be increased. In this case, the zeros and poles have been manually adjusted by inspection. A more elaborated and automatic procedure can be found in [11]. For the given sensor, the transfer function has been determined to be as described in Equation (7) with the following values: *α* = 3.78, *z*_1_ = 2π∙3.9 × 10^6^ rad/s, *z*_2_ = 2π∙130 × 10^6^ rad/s, *p*_1_ = 2π∙1.1 × 10^6^ rad/s and *p*_2_ = 2π∙9 × 10^6^ rad/s.

(7) $$H\left(s\right)=\frac{U\left(s\right)}{I\left(s\right)}=\frac{\alpha \cdot s\left(s+z_{1}\right)\left(s+z_{2}\right)}{{\left(s+p_{1}\right)}^{2}\left(s+p_{2}\right)}$$

The simulated frequency response according to Equation (7) is shown in Figure 4 along with the measured frequency response. Since both frequency responses are similar in magnitude and phase, the model shown in Equation (7) is therefore validated. 

## 3. Charge Estimation Theory

### 3.1. Charge Estimation Theory Using the Simplified HFCT Model

Considering Equation (1), it is possible to derive the calculation of the charge, *q*(*t*), knowing that the definition of charge is the integral of the current *i*(*t*). 

To calculate the evolution of charge over time, a double integration in the time domain is applied to Equation (1). Proceeding as described, Equation (1) can be written as follows.

(8) $$\underset{q\left(t\right)}{\underset{︸}{\iint \frac{di\left(t\right)}{dt}dt^{2}}}=\underset{\frac{L_{2}C}{M}u\left(t\right)}{\underset{︸}{\frac{L_{2}C}{M}\iint \frac{d^{2}u\left(t\right)}{dt^{2}}dt^{2}}}+\underset{\frac{L_{2}}{RM}\int u\left(t\right)dt}{\underset{︸}{\frac{L_{2}}{RM}\iint \frac{du\left(t\right)}{dt}dt^{2}}}+\frac{1}{M}\iint u\left(t\right)dt^{2}$$

Assuming no initial conditions and simplifying Equation (8), the final equation for *q*(*t*) is:

(9) $$q\left(t\right)=\underset{\begin{array}{c} first\\ term \end{array}}{\underset{︸}{\frac{L_{2}C}{M}u\left(t\right)}}+\underset{\begin{array}{c} second\;\\ term \end{array}}{\underset{︸}{\frac{L_{2}}{RM}\overset{t}{\underset{0}{\int }}u\left(t\right)dt}}+\underset{\begin{array}{c} third\\ term\; \end{array}}{\underset{︸}{\frac{1}{M}\overset{t}{\underset{0}{\iint }}u\left(t\right)dt^{2}}}$$

Considering that *i*(*t*) is a current pulse of limited duration, *u*(*t*) will tend to zero after the current pulse *i*(*t*) has been extinguished. Therefore, the first term of *q*(*t*) tends to zero when *t* tends to infinite. After a certain time, *t_int_*, the time integral of *u*(*t*) tends to zero, since an inductive measuring system does not measure the DC component, which in turn nulls the second term. Therefore, simplifying Equation (9), the total charge, *Q*, of the input current pulse *i*(*t*) can be calculated as follows.

(10) $$Q\approx q\left(t_{int}\right)\approx \frac{1}{M}\overset{t_{int}}{\underset{0}{\iint }}u\left(t\right)dt^{2}$$

This mathematical analysis reveals that, theoretically, any partial discharge sensor based on magnetically coupled inductors that can be modelled as a second-order differential equation can measure the input charge independently of sensor design and frequency response. According to the previous equation, the charge *Q* can be calculated via the double time integral of the measured voltage *u*(*t*), knowing the mutual inductance *M*. Similar results for Rogowski coils have been presented in [12].

### 3.2. Charge Estimation Theory Using the Generic HFCT Model

It has been shown that the simplified HFCT model has limited applicability depending upon the sensor frequency response. A generic model for HFCT transformers can be created by a combination of zeros and poles, as shown in Equation (11), if no complex poles are needed to model the frequency response of the sensor. The number and location of the zeros and the poles depend on the frequency response to be modelled.

(11) $$H\left(s\right)=\frac{U\left(s\right)}{I\left(s\right)}=\frac{\alpha \cdot s\cdot \sum_{i=1}^{i=m}\left(s+z_{i}\right)}{\sum_{j=1}^{j=n}\left(s+p_{j}\right)}$$

Using Equation (11), it is possible to express the double time integral of the measured voltage in the Laplace domain as follows.

(12) $$\frac{U\left(s\right)}{s^{2}}=I\left(s\right)\frac{\alpha \cdot \sum_{i=1}^{i=m}\left(s+z_{i}\right)}{s\cdot \sum_{j=1}^{j=n}\left(s+p_{j}\right)}$$

Applying a partial fraction expansion, it is possible to break Equation (12) down into simple terms expressed as a function of the poles and the coefficients *a_n_*.

(13) $$\;\frac{U\left(s\right)}{s^{2}}=I\left(s\right)\left(\frac{a_{0}}{s}+\frac{a_{1}}{\left(s+p_{1}\right)}+\ldots +\frac{a_{n}}{\left(s+p_{n}\right)}\right)$$

Using the inverse Laplace transform, Equation (13) can be written in the time domain as follows.

(14) $$\overset{t}{\underset{0}{\iint }}u\left(t\right)dt^{2}=a_{o}\underset{q\left(t\right)}{\underset{︸}{\overset{t}{\underset{0}{\int }}i\left(t\right)dt}}+\overset{i=n}{\underset{i=1}{\sum }}\underset{addends}{\underset{︸}{a_{i}\cdot \underset{\to 0\;as\;t\to \infty }{\underset{︸}{e^{-p_{i\cdot }t}}}\cdot \overset{t}{\underset{0}{\int }}e^{p_{i\cdot }t}\cdot i\left(t\right)dt}}\;$$

Equation (14) shows that for any arbitrary model based on the generic model of the HFCT, the value of the double time integral contains information about the charge of the current pulse. 

Since a partial discharge current pulse has a limited duration, the integrals in the addends reach a constant value after the time duration of the current pulse. Once an integral has reached its constant value, it is damped by the exponential decay with the time constant determined by its pole. Hence, over time, all the addends in the summation tend to zero, and therefore the summation. The speed of the convergence of the summation to zero is dominated by the smaller pole, named *p*_1_, which imposes the slowest dynamic. Assuming that the duration of the current pulse is shorter than the time constant determined by *p*_1_, the total charge *Q* could be approximated by

(15) Q ≈∬04p1u(t)dt2a0

An integration time of *4/p*_1_ has been selected, since the first exponential decay has already decreased by 98% of its initial value at this moment in time. Therefore, *4/p*_1_ is a good estimation of the necessary integration time for current pulses with a pulse duration shorter than it. 

This result proves that the double time integral of the measured voltage is proportional to the total charge of the current pulse after a certain integration time. This statement is valid irrespective of the sensor frequency response (determined by the number of zeros and poles), provided that the sensor exhibits a derivative behavior in the low-frequency range. This required characteristic is typical of sensors based on magnetically coupled inductors, which applies to HFCT sensors, Rogowski coils and to the recently developed magnetic loop antennas used to measure partial discharges in gas-insulated systems (GISs) [13,14].

According to Equation (15), to estimate the charge value, the first constant value *a_0_* of the partial fraction expansion shown in Equation (13) must be determined. Using Equation (13), it is possible to rewrite the transfer function of the sensor as follows.

(16) $$\frac{U\left(s\right)}{I\left(s\right)}=a_{0}\cdot s+\frac{a_{1\cdot }s^{2}}{\left(s+p_{1}\right)}+\ldots +\frac{a_{n}\cdot s^{2}}{\left(s+p_{n}\right)}$$

In the frequency domain, Equation (16) is equivalent to the following equation.

(17) $$\;\frac{U\left(\omega \right)}{I\left(\omega \right)}=j\omega a_{0}-\frac{a_{1\cdot }\omega^{2}}{\left(p_{1}+j\omega \right)}-\ldots -\frac{a_{n}\cdot \omega^{2}}{\left(p_{n}+j\omega \right)}$$

When ω <<< *p_1_*, then *(p_i_ + j* ω*) ≈ p_i_*. Hence, Equation (17) can be simplified as follows

(18) $$H\left(\omega \right)=\frac{U\left(\omega \right)}{I\left(\omega \right)}\approx j\omega a_{0}-\frac{a_{1\cdot }\omega^{2}}{p_{1}}-\ldots -\frac{a_{n}\cdot \omega^{2}}{p_{n}}$$

By applying the derivative with respect to ω in Equation (18), the following result applies

(19) $$\frac{dH\left(\omega \right)}{d\omega }\approx \left(ja_{0}-\frac{2a_{1\cdot }\omega }{p_{1}}-\ldots -\frac{2a_{n}\omega }{p_{n}}\right)$$

Therefore, when ω tends to zero, the following result holds for the derivative of the transfer function.

(20) $${\frac{dH\left(\omega \right)}{d\omega }|}_{\omega \to 0}\approx ja_{0}$$

This result means that the slope of the Bode magnitude plot is a good approximation of the value of *a_o_*. Since the derivative of *H*(ω) when ω tends to zero is a complex number, the value of the slope must be determined in the low-frequency ranges, where the Bode phase plot is close to 90°.

**Figure 6:**
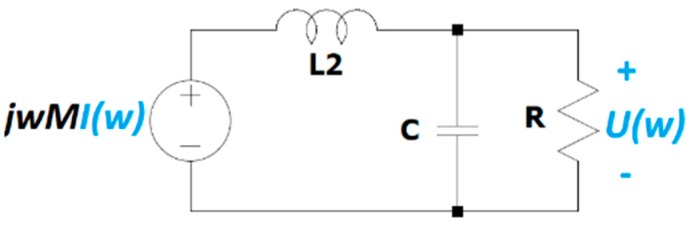
Equivalent circuit in the Laplace domain of the secondary coil of the magnetically coupled sensor.

A circuital analysis of the HFCT basic circuit helps to give this slope a physical meaning. Figure 6 shows the equivalent circuit of the secondary of a HFCT in the frequency domain.

In the low-frequency range, when ω tends to zero, the impedance of the inductor tends to zero and the impedance of the capacitor to infinite. Therefore, the voltage that appears across the resistor equals the induced voltage in the secondary coil of the magnetic coupling. Under this condition, the transfer function can be approximated by the following equation.

(21) $$H\left(\omega \right)=\frac{U\left(\omega \right)}{I\left(\omega \right)}\approx j\omega M\;\;\;\;\;when\;\;\omega \to 0\;rad/s$$

Equation (21) reveals that the value of *a*_0_ matches the mutual inductance *M*. The mutual inductance can then be experimentally determined by the slope of the Bode magnitude plot in the low-frequency range, where the Bode phase plot is 90°. This result, obtained for a generic HFCT model, is in accordance with Equation (10), which was obtained for the simplified model using electrical parameters, thus demonstrating that the simplified model is a simple particular case of the generic model. 

## 4. Charge Estimation Methods

The charge evolution over time and the integration time strongly depends on the inductive sensor characteristics as defined in Equation (14). As explained in Section 3.2 and shown in Equation (15), the double time integral of the measured voltage converges to the charge value after a certain integration time. When the duration of the current pulse is smaller than the time constant determined by *4/p*_1_, the integration time can then be approximated by *4/p*_1_, *p*_1_ being the first pole of the transfer function. An estimation of *p_1_* can be calculated using the sensor parameters as *R/L*_2_. 

Table 3 shows the estimated integration time using both methods for the HFCT_LG and HFCT_HG sensor. 

To illustrate the convergence of the double time integral, a 5 pC triangular pulse of 10 ns duration has been used to simulate the HFCT responses to the current pulse. 

The responses of the sensors have been simulated using the transfer functions defined in Section 2.1 and Section 2.2, using the zeros and poles. The mutual inductance *M* has been estimated by the slopes of the Bode magnitude plots between 1 kHz and 4 kHz, since both Bode phase plots show phase values close to 90° between these two frequencies; see Figure 3 and Figure 4. The estimated value of *M* for the HFCT_LG sensor is 24.4 µH, and 28 µH for the HFCT_HG sensor.

**Figure 7:**
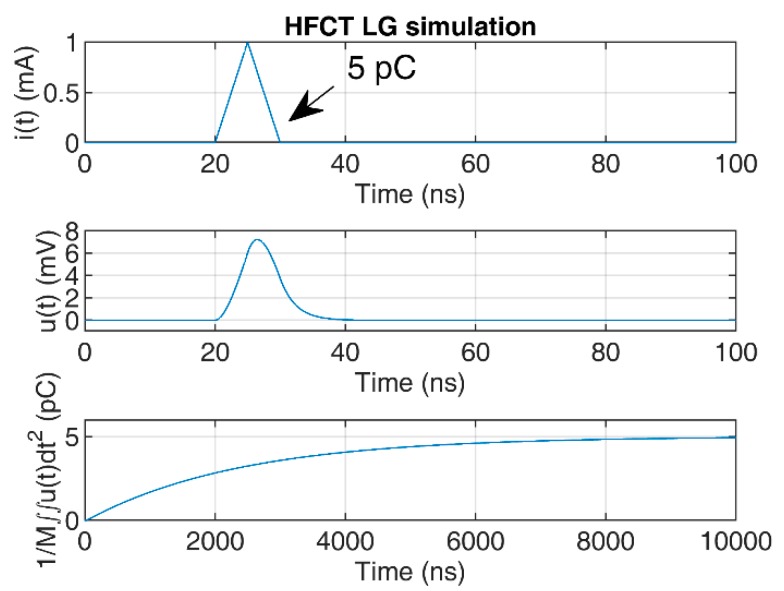
HFCT_LG sensor response and charge estimation of a 5 pC and 10 ns triangular current pulse.

**Figure 8:**
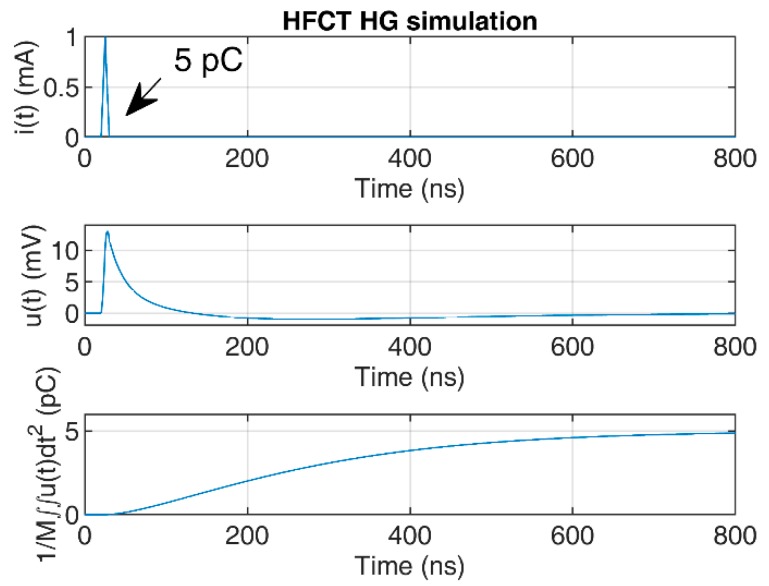
HFCT_HG sensor response and charge estimation of a 5 pC and 10 ns triangular current pulse.

Figure 7 and Figure 8 show the current pulse, the simulated voltage outputs and the charge evolution. In this case, since the current pulse duration is smaller than the estimated integration times shown in Table 3, the convergence of the charge estimation approaches the charge value within the estimated integration times. 

A longer current pulse duration has been simulated to illustrate the effects on the convergence of the double time integral for the charge estimation. 

**Figure 9:**
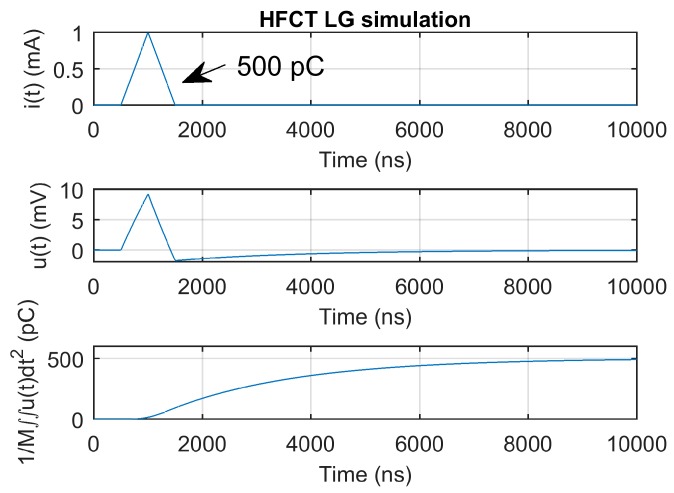
HFCT_LG sensor response and charge estimation of a 500 pC and 1000 ns triangular current pulse.

Figure 9 shows the HFCT_LG response and charge estimation to a 500 pC and 1000 ns current pulse. Since the current pulse duration is still smaller than the estimated integration time of 9.5 µs, the convergence of the double time integral of the voltage falls within the expected integration time.

**Figure 10:**
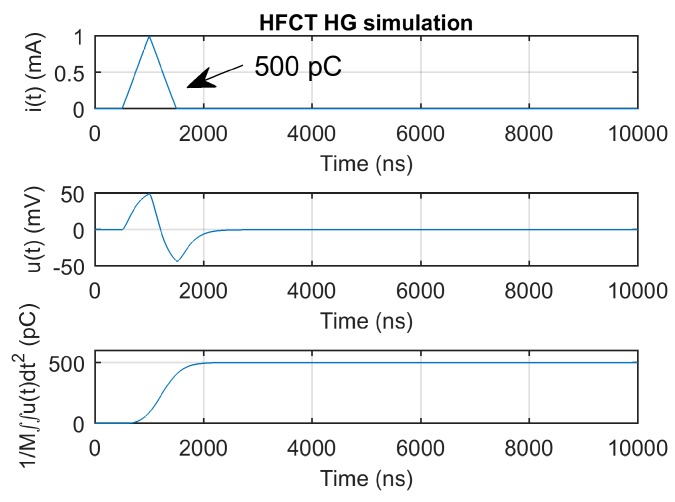
HFCT_HG sensor response and charge estimation of a 500 pC and 1000 ns triangular current pulse.

However, the 1000 ns long triangular current pulse has a pulse duration that is almost twice the estimated integration time of the HFCT_HG sensor, as indicated in Table 3. As shown in Figure 10, the required integration time is approximately 2000 ns, which matches the voltage pulse duration. For that reason, in partial discharge measurements, where the current pulse duration is expected to be bigger than the integration time, as calculated by the first pole of the transfer function, it is advisable to estimate the integration time via the analysis of the measured voltage. The estimated integration time could, in this case, be approximated by the time between the starting and the second zero crossing of the measured voltage signal.

It is worth noticing that for the HFCT_LG sensor, the integration time is remarkably longer than the current pulse duration, since the lower cutoff frequency is in the kHz range. Moreover, for this particular sensor, the undershoot produced in the measured voltage is very small. In practice, this could lead to integration errors due to offsets and the vertical scale digitalization resolution. Moreover, the longer the required integration time, the higher the probability to be affected by noise or by external disturbances. Therefore, if the sensor requires a long integration time using the double time integral method, but the sensor has a broadband flat frequency response that allows for a reliable current pulse shape measurement, then an alternative method for charge estimation is to estimate the current pulse using the sensor gain and the measured voltage. Once the current is estimated, the charge can be evaluated by the time integration between the voltage signal zero crossings, as explained in [15].

## 5. Test Measurements

Two cases of study are presented to illustrate the principles of the charge estimation methods and the calibration methods presented before. For that purpose, the HFCT_LG sensor and the HFCT_HG sensor are used. A partial discharge calibrator is used to inject a nanosecond current pulse and to test the performance of each method.

### 5.1. Sensor Characterization

**Figure 11:**
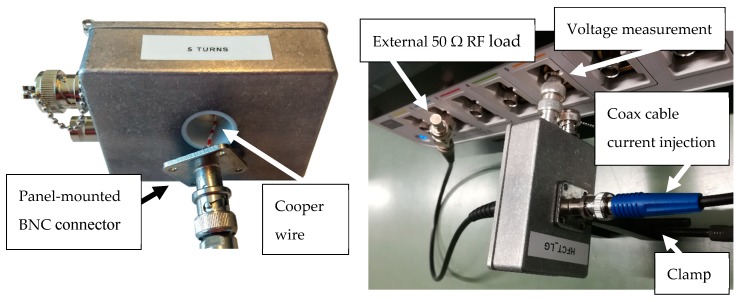
HFCT testing setup and sensor connection to the oscilloscope.

To characterize the HFCT sensors, they have been provided with two panel-mounted BNC connectors, one in each side of the enclosure, internally connected using a copper wire and fixed to the enclosure using a clamp; see Figure 11. 

The setup allows a continuous coaxial arrangement in which a voltage source or a partial discharge calibrator is connected to one BNC connector, and the other BNC connector to an oscilloscope to measure the injected signals. In this case, an external 50 Ω resistor is connected to a 1 MΩ and 500 MHz bandwidth input channel of an oscilloscope. The injected currents are evaluated as the measured voltage over the 50 Ω external RF load resistance. The HFCT sensor output is directly connected to a 1 MΩ and 500 MHz bandwidth input channel of the oscilloscope.

The same arrangement is used with both sensors to determine its frequency response using a signal generator, and later to inject calibrator PD signals to check each of the charge estimation methods. This arrangement is similar to the one described in [16].

### 5.2. Case Study: HFCT_HG

This case study uses a HFCT with the parameters described in Table 2. The frequency response of the sensor is shown in Figure 4. According to Table 3, the integration time for this sensor is approximately 578 ns. Since the sensor has a short integration time, it is decided to use the double time integral to estimate the charge.

**Figure 12:**
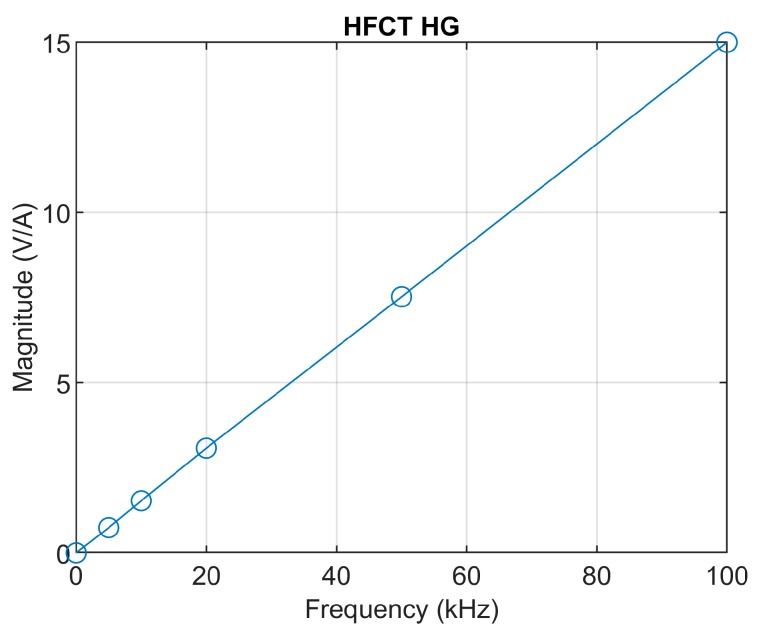
HFCT_HG sensor low-frequency magnitude response.

A detailed Bode magnitude plot at low frequencies is depicted in Figure 12. The frequency response of the sensor clearly shows a linear behavior in the low-frequency range below 100 kHz. The mutual inductance is therefore calculated as the slope of the magnitude frequency response, accounting for 15/(2π∙100 × 10^3^) ≈ 2.38 × 10^−5^ H.

**Figure 13:**
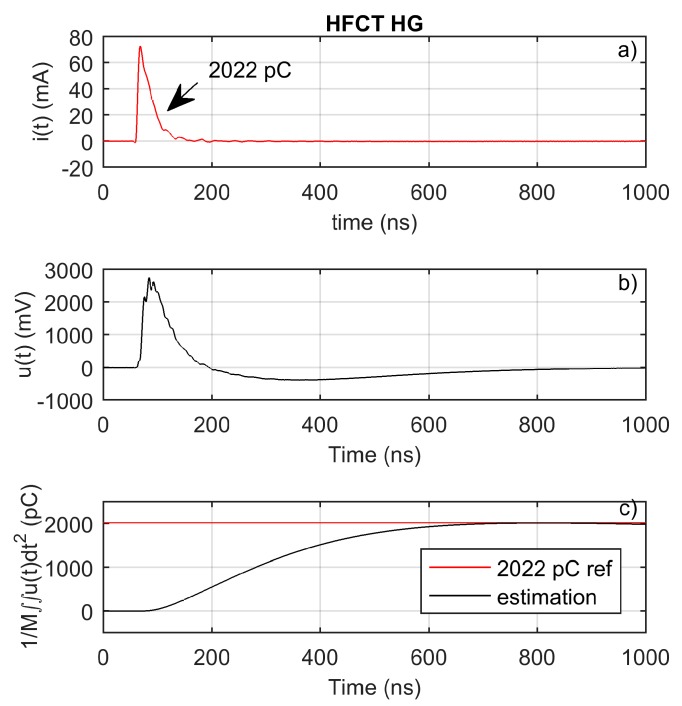
HFCT_HG: (**a**) injected calibrator current pulse; (**b**) measured pulse; (**c**) charge estimation using double integral.

A calibration pulse has been injected to validate the charge estimation method. The injected pulse is shown in Figure 13a. Figure 13b shows the measured voltage. Figure 13c clearly shows that the charge estimation method using the double time integral and the mutual inductance is a suitable charge estimation method for this HFCT sensor. 

### 5.3. Case Study: HFCT_LG

This case study uses a HFCT with the parameters described in Table 1. The frequency response of the sensor is shown in Figure 3. According to Table 3, the integration time for this sensor is approximately 9.4 µs. Since the sensor has a long integration time but a flat and broadband frequency response, it is decided to estimate the charge using the sensor gain and the first integral of the measured voltage. In this case, the charge is evaluated as

(22) $$Q=\overset{\infty }{\underset{0}{\int }}i\left(t\right)dt\approx \frac{1}{H_{max}}\overset{t_{zc2}}{\underset{t_{zc1}}{\int }}u\left(t\right)dt$$

where *H_max_* is the sensor gain, equal to 10.3 mV/mA, and *t_zc_*_1_ and *t_zc_*_2_ are the *u*(*t*) zero-crossing times. 

The sensor has been used to measure a calibrator pulse. The injected charge is obtained by direct integration of the current through the oscilloscope input impedance. The injected charge is of 2022 pC. 

**Figure 14:**
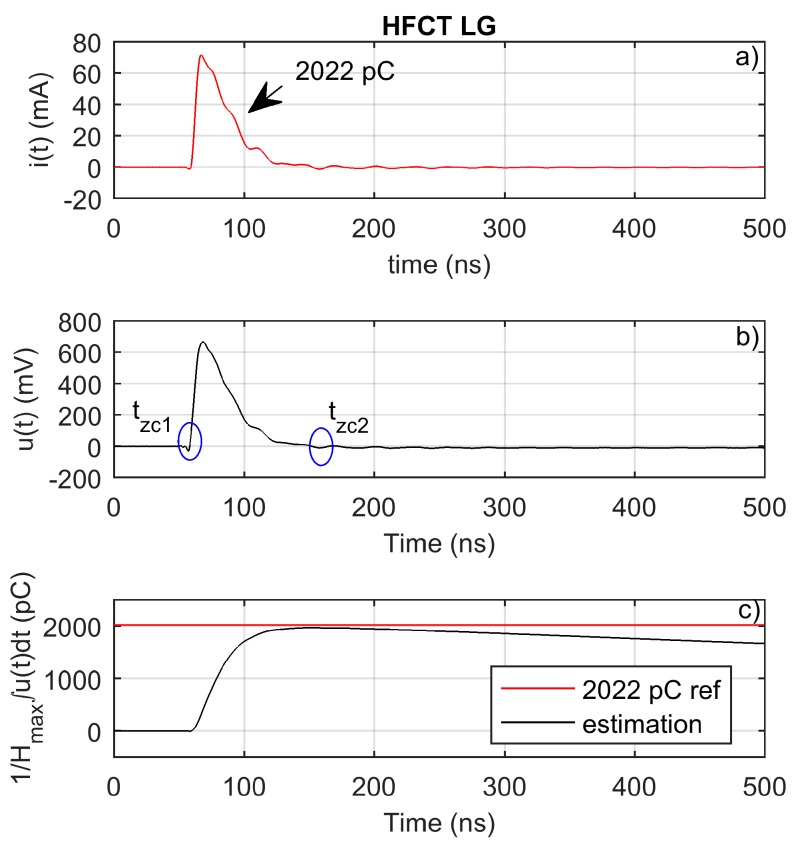
HFCT_LG: (**a**) injected calibrator current pulse; (**b**) measured pulse; (**c**) charge estimation using peak integration.

Figure 14b depicts the measured voltage pulse. As shown, the measured pulse shape is very accurate, with almost no pulse distortion. The measured pulse has an almost unnoticeable low-level and long-lasting pulse undershoot. This undershoot is responsible for the integral value decrease after *t_zc2_*—see Figure 14c—which will finally reach zero. This behavior is due to the broadband and flat frequency response of the sensor. Moreover, the peak value of the integral occurs at nearly the same time as the current pulse has been extinguished. 

Figure 14c shows how the peak of the first time integral of the measured voltage approximated the injected charge with a small error, therefore validating the charge evaluation method for this sensor. In this case, the peak value of the time integral is equal to the integral value between the zero crossings of the measured voltage signal.

## 6. Discussion on HFCT Sensor Design Considerations

The design choices, constructive aspects and magnetic material properties of a HFCT sensor determine and shape its frequency response. 

According to the simplified model of a HFCT, when the lower cutoff frequency, *f_−3db_low_*, and the higher cutoff frequency, *f_−3db_high_*, are separated enough, the maximum gain, *H_max_*, and the cutoff frequencies can be estimated by:

(23) $$f_{-3db_{low}}=\frac{R}{2\pi L_{2}}\;\;\;\;\;\;\;f_{-3db_{high}}=\frac{1}{2\pi RC}\;\;\;\;\;\;\;H_{max}=\frac{RM}{L_{2}}$$

**Figure 15:**
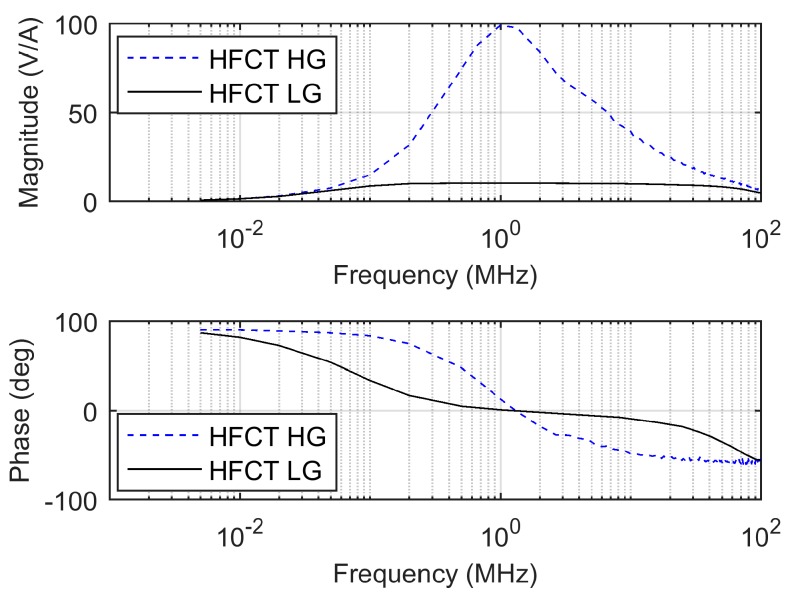
Comparison of Bode plots of the HFCT_LG and the HFCT_HG sensors.

As shown, for a given secondary inductance and capacitance (accounting for the added capacitor and parasitic capacitances), an increase in the load resistance increases the maximum gain, increases the lower cutoff frequency, and decreases the higher cutoff frequency. In practice, this is translated into a peaky frequency response that heavily distorts the pulse shape, since the frequency components of the current pulse are amplified and phase shifted at different levels. This phenomenon can be appreciated in the comparison of the frequency responses of the HFCT_LG and the HFCT_HG sensors shown in Figure 15. 

Due to the physical relationships between *f_−3db_low_*, *f_−3db_high_*, and *H_max_*, it is impossible to optimize one characteristic of the sensor without affecting the other ones. In practice, this means that a HFCT sensor with a flat and broadband frequency response will have a smaller *H_max_* gain than a HFCT sensor with a non-flat frequency response.

Regarding partial discharge measurements, a bigger *H_max_* gain is an interesting property, since it increases the measured voltage peak values. Table 4 shows the ratio of the measured voltage peaks to the same current pulses when using the HFCT_LG and the HFCT_HG sensors.

However, the higher peak voltages measured with the HFCT_HG sensor are at the expense of pulse shape accuracy, which jeopardizes the extraction of any pulse shape-related feature, such as rise time and tail time, or charge estimation using the first time integral of the measured voltage. 

This paper has demonstrated that one important current pulse feature—that is, the charge of the current pulse—can be properly estimated using the double time integral of the measured voltage, and, moreover, that the calibration constant can be obtained from the frequency response of the sensor. This statement is valid irrespective of the frequency response of the HFCT sensor, assuming that its transfer function can be modelled as a combination of real zeros and poles.

From a practical point of view, this result leads to the conclusion that in partial discharge measurements where the pulse shape accuracy is not a relevant factor, the sensitivity of the measurement can be increased by designing a high-gain HFCT sensor. Along with the gain, the expected integration times must be then considered in the design depending upon the partial discharge expected time duration. Current pulse durations can be in the tens of nanosecond range in compact partial discharge-measuring test setups or in GISs, and in the microsecond range in electrical machines or long cables. Therefore, design choices are needed depending upon the application.

## 7. Conclusions

This paper shows a simplified and a generic model of HFCT sensors. Using the generic model, the principles of charge evaluation when using inductive sensors based on magnetically coupled inductors in partial discharge measurements have been explained.

The mathematical analysis of the generic transfer function model reveals that, theoretically, the double time integral of the measured voltage is an exact method for charge estimation irrespective of the sensor frequency response and characteristics. Moreover, the calibration of the sensor for charge estimation purposes can be carried out using the Bode plots of the sensor. The practical limitations of this charge estimation method rely on the necessary integration times.

Depending upon the application, the expected current pulse duration and the sensor, a suitable charge estimation method must be selected. 

The charge estimation method based on the first integral of the measured voltage proves to be a suitable method when the current pulse waveform is accurately measured. This is normally the case when a HFCT sensor with a flat and broadband frequency response is used. In this case, any other pulse shape-related features, such as rise time and tail time, can also be properly estimated.

The charge estimation method based on the evaluation of the double time integral of the measured voltage is a suitable method when the sensor has an expected short integration time.

Since both methods are based on integration, it is important to determine the integration limits to minimize the contribution of signal offsets and noise in the charge evaluation. Therefore, it is recommended to use the voltage zero-crossing points as integration limits. 

## Figures and Tables

**Table 1:** HFCT_LG sensor parameters.

Sensor	HFCT Sensor Parameters			
	L_1_ (μH)	L_2_ (μH)	C (pF)	R (Ω)
HFCT_LG	5.25	130.64	28.26	55.61

**Table 2:** HFCT_HG sensor parameters.

Sensor	HFCT Sensor Parameters			
	L_1_ (μH)	L_2_ (μH)	C (pF)	R (Ω)
HFCT_HG	4.90	127.14	26.65	996.4

**Table 3:** Estimation of integration times for charge calculation using the double time integral of the measured voltage.

**Estimated integration times for current pulse durations <<< *4/p*_1_**	**HFCT_LG**		**HFCT_HG**	
	*4/p*_1_	*4∙L*_2_ */R*	*4/p*_1_	*4∙L*_2_ */R*
	9.5 µs	9.4 µs	578 ns	510 ns

**Table 4:** Ratio of peak values for the same current pulses using the HFCT_LG and HFCT_HG sensors.

Charge (pC)	Pulse Duration (ns)	Current Pulse Shape	$\frac{{V_{peak}|}_{HFCT\_HG}}{{V_{peak}|}_{HFCT\_LG}}$
5	10	Triangular (Figure 7, Figure 8)	≈1.8
500	1000	Triangular (Figure 9, Figure 10)	≈5.5
2022	100	Calibrator (Figure 13, Figure 14)	≈4.1
