# Erratum: Rodrigo-Mor et al. Principles of Charge Estimation Methods Using High-Frequency Current Transformer Sensors in Partial Discharge Measurements. *Sensors* 2020, *20*, 2520

**DOI:** 10.3390/s21186010

**Published:** 2021-09-08

**Authors:** Armando Rodrigo-Mor, Fabio A. Muñoz, Luis Carlos Castro-Heredia

**Affiliations:** Electrical Sustainable Energy Department, Delft University of Technology, 2628 CD Delft, The Netherlands; f.a.munozmunoz-1@tudelft.nl (F.A.M.); L.C.CastroHeredia@tudelft.nl (L.C.C.-H.)

The authors wish to make the following erratum to this paper [1]: the summation symbol in the Equations (11) and (12) should be a product symbol. 

The corrected Equations (11) and (12) appear below:

(11) $$H\left(s\right)=\frac{U\left(s\right)}{I\left(s\right)}=\frac{\alpha \cdot s\cdot \prod_{i=1}^{i=m}\left(s+z_{i}\right)}{\prod_{j=1}^{j=n}\left(s+p_{j}\right)}$$

(12) $$\frac{U\left(s\right)}{s^{2}}=I\left(s\right)\frac{\alpha \cdot \prod_{i=1}^{i=m}\left(s+z_{i}\right)}{s\cdot \prod_{j=1}^{j=n}\left(s+p_{j}\right)}$$

The authors apologize for any inconvenience caused and state that the scientific conclusions are unaffected. The original article has been updated.
